# The Healthy Diet Basket is a valid global standard that highlights lack of access to healthy and sustainable diets

**DOI:** 10.1038/s43016-025-01177-0

**Published:** 2025-05-27

**Authors:** Anna W. Herforth, Yan Bai, Aishwarya Venkat, William A. Masters

**Affiliations:** 1 Food Prices for Nutrition Project, Friedman School of Nutrition Science and Policy, Tufts University, Boston, MA USA; 2 Development Data Group, The World Bank, Washington, DC USA; 3 School of Public Affairs, Zhejiang University, Hangzhou, China

**Keywords:** Social policy, Developing world, Economics, Sustainability

## Abstract

The Healthy Diet Basket (HDB) is a standard developed from food-based dietary guidelines (FBDG) for the measurement of the Cost and Affordability of a Healthy Diet—a new indicator of food security tracked by the United Nations Food and Agriculture Organization and the World Bank. Here we analysed the HDB’s economic, nutritional and environmental characteristics of least-cost diets relative to 16 national FBDG and the EAT-Lancet reference diet. The HDB cost averaged US$3.68 per person per day in 2021, slightly lower than most FBDG. Macronutrient levels fell within acceptable macronutrient distribution ranges, and the average mean adequacy ratio of 15 micronutrients and protein was 95% for the HDB, equivalent to the average mean adequacy ratio across FBDG. The HDB’s carbon and water footprints were found to be similar to the EAT-Lancet reference diet. These findings demonstrate the use of the HDB as a global standard and highlight the lack of access to healthy and sustainable diets globally.

## Abstract (web summary)

The Healthy Diet Basket (HDB) is used for the measurement of Cost and Affordability of a Healthy Diet globally. This analysis compares the economic, nutritional and environmental characteristics of least-cost diets following the HDB with national food-based dietary guidelines and the EAT-Lancet diet, demonstrating the use of the HDB as a global standard of healthy and sustainable diets.

## Main

Access to adequate food is arguably the most basic need. It was the impetus for international collaboration and the creation of the Food and Agriculture Organization (FAO), which today remains the custodian of global food security measurement, reflecting its founding ideals. Since 1996, food security has been defined as ‘access to sufficient, safe, nutritious food to meet dietary needs and food preferences for an active and healthy life’—in short, access to a healthy diet^1^. This aspiration has been measured for decades using the prevalence of undernourishment, an indicator of dietary energy, and more recently using indicators of moderate and severe food insecurity based on the Food Insecurity Experience Scale^2^. Neither one, however, fully captures access to healthy diets.

The Cost and Affordability of a Healthy Diet (CoAHD) indicator was developed to measure economic access to nutritious food to meet dietary needs, joining the prevalence of undernourishment and Food Insecurity Experience Scale-based indicators to more fully reflect the United Nations (UN) definition of food security^3^. It has been reported by FAO and other UN agencies annually since 2020 (ref. ^4^). The cost of a healthy diet (CoHD) is the retail cost of purchasing the least expensive locally available foods to meet food-based dietary guidelines (FBDG)^5,6^. This cost is then compared to income available for food to determine affordability^7,8^. The objective of the CoAHD as an indicator of economic access to adequate food is to define a price floor, below which it is not possible to purchase a healthy diet. The indicator is solely based on observable prices in the market and not on consumption behaviour which is influenced by many factors in addition to prices (such as tastes, preferences, attitudes, knowledge and time).

A healthy diet is defined as one that meets FBDG, which are formulated within national contexts to define a dietary pattern that meets nutrient needs, protects health against diet-related non-communicable diseases and accommodates cultural, religious and personal preferences, as well as local availability and affordability of items within food groups. For national applications of CoAHD, FBDG are an appropriate healthy diet standard, coherent with social assistance and nutrition education programs based on the FBDG. For global monitoring and comparisons, it is necessary to use a single comparable standard. The ‘Healthy Diet Basket’ (HDB) was developed to meet this need, and it has been used as a cost standard for global monitoring of CoAHD since 2022^5,9^.

The HDB was constructed to reflect the commonalities in national FBDG around the world^9^. It is a set of six food groups (starchy staples, vegetables, fruits, animal-source foods, legumes, nuts and seeds, and oils and fats) in the average proportions of each food group recommended across a range of quantified and semi-quantified FBDG (Table 1). These can be populated with any items that belong in the food group, but a minimum level of diversity is required in each group, in keeping with the principle universally expressed in FBDG to consume a diversity of foods. When applied in CoAHD, a minimum number of least-cost items (11) are identified using price data, and the cost of the amounts needed to meet the specified energy content of each food group is calculated. The total energy content of the HDB is 2,330 kcal, which represents the energy needs of a representative person in the population based on the dietary energy requirement of an active 30-year-old woman, approximately equivalent to the average energy requirements across age and population groups^9,10^.

**Table 1:** HDB content by food group

Food group	Total energy content (kcal)	Percentage of dietary energy	Minimum number of food items selected for CoAHD
**Starchy staples**	1 160	50%	2
**Vegetables**	110	5%	3
**Fruits**	160	7%	2
**Animal source foods**	300	13%	2
**Legumes, nuts and seeds**	300	13%	1
**Oils and fats**	300	13%	1

This study aims to validate the HDB as a cost standard for a healthy and sustainable diet, by assessing whether its nutritional characteristics are comparable to national FBDG and whether its environmental impact is comparable to the EAT-Lancet reference diet, introduced in 2019 by the EAT-Lancet Commission as a healthy dietary pattern designed for environmental sustainability^11^. Using 2021 national food price data for 173 countries from the International Comparison Program (ICP), we compare the economic, nutritional and environmental sustainability characteristics of least-cost diets following HDB to 16 recent, quantifiable national FBDG in diverse regions throughout the world^12–27^ and the EAT-Lancet reference diet^11^.

## Results

### CoAHD

Least-cost baskets of items meeting the criteria for each of the 16 FBDG, the HDB and the EAT-Lancet reference diet were identified for 162–172 countries in 2021, resulting in a total of 3,071 baskets calculated.

**Fig. 1:**
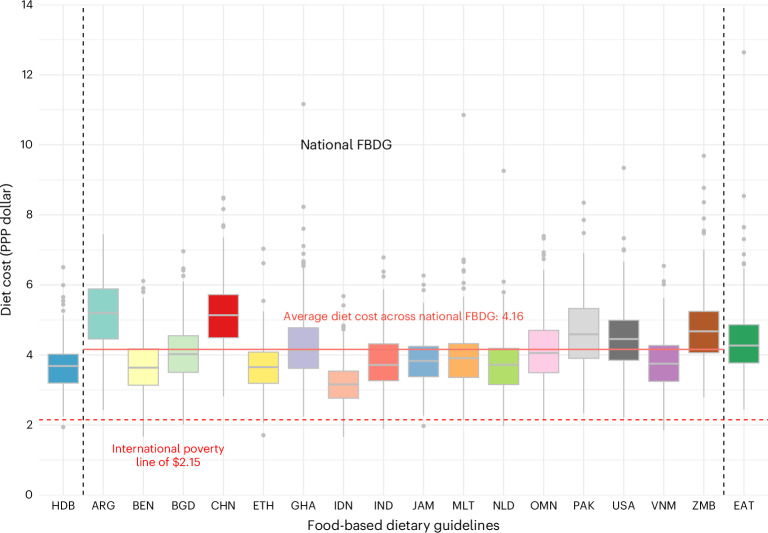
Cost of healthy diets. The cost of each recommended diet, defined as the cost of the least expensive food items to meet FBDG at a constant energy total of 2,330 kcal d^−^^1^ in 2021 PPP dollars per person per day. The box plots illustrate the distribution of diet costs across countries. The central horizontal line within each box represents the median cost, while the upper and lower edges of the box indicate the IQR, covering the 25th to 75th percentiles of country-level diet costs. The whiskers extend to the minimum and maximum values within 1.5 times the IQR, with individual points beyond this range representing outliers. Diet cost estimates were derived for 172 countries where available to calculate the cost of meeting FBDG of Argentina (ARG), Benin (BEN), Malta (MLT), Pakistan (PAK), the United States (USA) and Viet Nam (VNM); from 171 countries of the HDB, Bangladesh (BGD), China (CHN), Ghana (GHA), India (IND), Indonesia (IDN), Jamaica (JAM), the Netherlands (NLD) and Zambia (ZMB); from 169 countries of Ethiopia (ETH), Oman (OMN); and 162 countries of the EAT-Lancet reference diet (EAT). The variation in the number of countries is due to differences in price data availability for the specific food groups required by each guideline. In some countries, price data for items in certain food groups may be unavailable, leading to slight differences in country coverage across the various diet cost calculations. The red line highlights the mean of diet costs across 16 national FBDG, which was $4.16 (median, $4.03) in 2021. This is higher than the mean and median of the HDB ($3.68) but lower than EAT (mean, $4.48; median, $4.27). The red dashed line represents the international extreme poverty line of $2.15 in 2017 PPP dollars.

The average CoHD using the HDB was $3.68 per person per day in 2021 purchasing power parity (PPP) dollars (s.d., $0.75; interquartile range (IQR)), $3.20–4.02). The total cost was slightly lower than the average cost of national FBDG or the EAT-Lancet reference diet (Fig. 1). By any definition, the CoHD is higher than the international extreme poverty line of $2.15 defined by the World Bank, let alone the portion of the poverty line that could be credibly reserved for food.

**Fig. 2:**
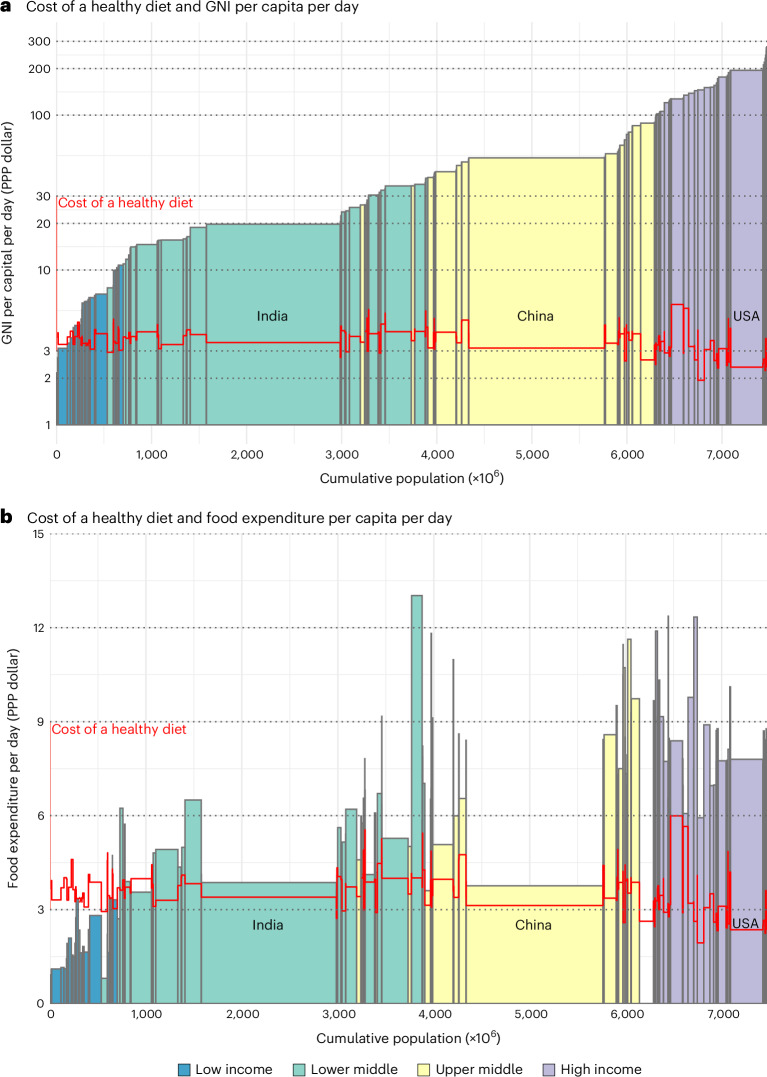
CoHD compared to GNI or food expenditure. **a**, GNI in 2021 PPP dollars per capita per day in a country, shown as the height of a bar, and the population as the width of the bar. **b**, Food expenditure in 2021 PPP dollars per capita per day in a country, shown as the height of a bar and the population as the width of the bar. Food expenditure data are missing for Russia. The red line indicates the CoHD, using the HDB.

**Extended Data Fig. 1:**
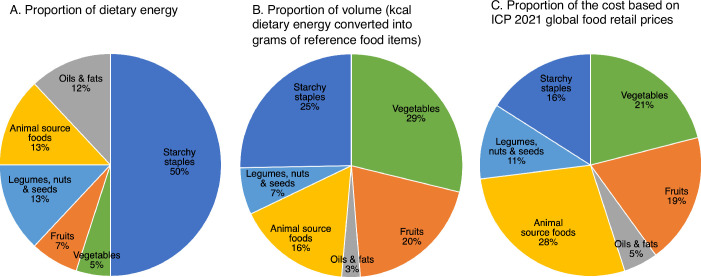
Composition of the Healthy Diet Basket. Composition of the Healthy Diet Basket by food group, displayed by (**A**) dietary energy contribution, (**B**) volume in grams using reference foods, and (**C**) cost contribution using global average prices from the ICP 2021 dataset. To equate calories and grams, the following reference food items were used for (**B**): dry rice for starchy staples, egg for animal source foods, dry bean for legumes/nuts/seeds, and average kcal/g across all non-duplicate items in the ICP 2017 dataset for fresh fruits and vegetables.

The CoHD in PPP dollars does not vary systematically by per capita gross national income (GNI) or food expenditure per capita (Fig. 2a,b). In almost all low-income countries, where a substantial portion of the global population resides, average food expenditures are below the CoHD. In most middle-income countries, which account for the largest portion of the global population, average food expenditures are around the CoHD, while in all high-income countries, average food expenditures are well above the CoHD. On average, globally, starchy staples accounted for 16% of the cost; oils 5%; legumes, nuts and seeds 11%; animal source foods 28%; and fruits and vegetables 40% (vegetables 21%, fruits 19%) (Extended Data Fig. 1).

### Nutritional content and environmental impacts of least-cost healthy diets

**Fig. 3:**
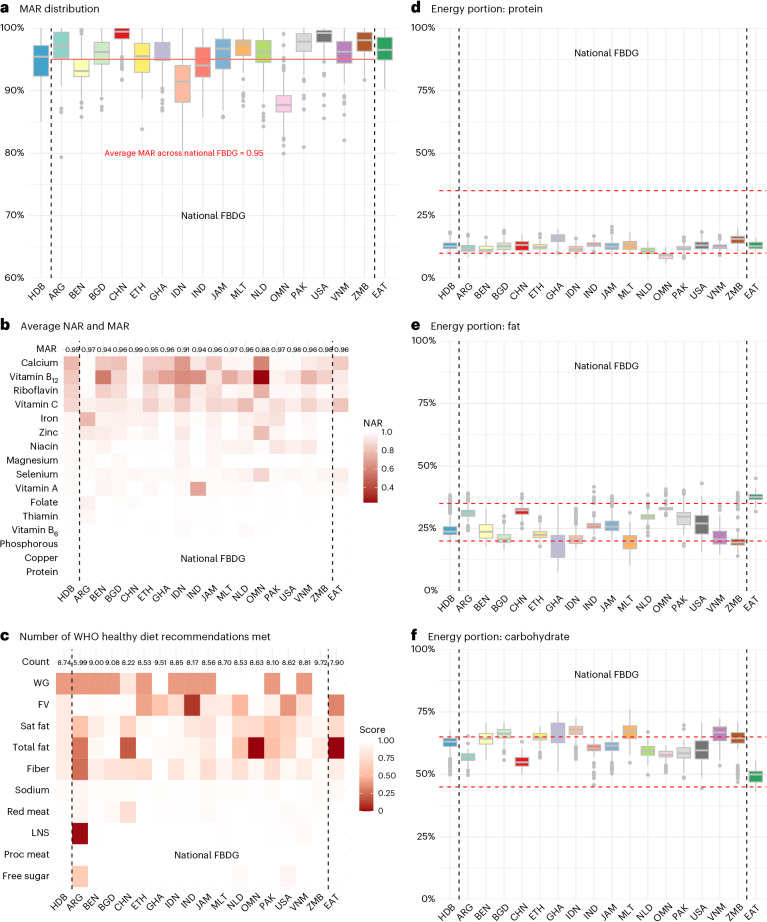
Nutrient adequacy, macronutrient distribution and compliance with WHO healthy diet recommendations. **a**, Assessment of nutrient adequacy and macronutrient distribution of least-cost diets by FBDG. This panel illustrates the distribution of MARs by FBDG, highlighting the mean MAR across all observations of national FBDG with a solid red line at 95% (median, 96%), at the same level as the HDB (mean and median, 95%) and the EAT-Lancet reference diet (mean, 96% and median, 97%). **b**, Average NARs for 15 essential micronutrients and protein, alongside the average MAR for each FBDG. **c**, Proportion of least-cost diets that meet WHO healthy diet recommendations by FBDG. WHO healthy diet recommendations include daily consumption of (1) 400 g or more fruits & vegetables (FV), (2) legumes, nuts & seeds (LNS), (3) whole grains (WG), (4) at least 25 g dietary fibre, (5) 30% or less of dietary energy from total fat, (6) 10% or less of dietary energy from saturated fat (sat fat), (7) less than 5 g of salt (2,000 mg sodium), (8) less than 10% of dietary energy from free sugar, (9) little or no processed meat (proc meat), and (10) less than 350–500 g per week unprocessed red meat (less than or equal to 71 g per day in the calculation). The number of recommendations met is counted, where each component is counted as 1 or 0. The mean count across national FBDG is 8.63 out of 10, lower than 8.74 for HDB but higher than 7.91 for EAT. **d**–**f**, The distribution of dietary energy contribution from three macronutrients (protein (**d**), total fat (**e**) and carbohydrate (**f**)). The red dashed lines indicate the upper and lower bounds of the AMDR. For adults, the AMDR is 10–35% of total dietary energy from protein, 20–35% from total fat and 45–65% from carbohydrates (Institute of Medicine 2006). Box plots in **a** and **d**–**f** show the IQR, with the horizontal line inside each box representing the median. Whiskers extend to the smallest and largest observations within 1.5 times the IQR, and individual points beyond this range are outliers.

For micronutrients and protein, the mean adequacy ratio (MAR) of the HDB was 95% (s.d., 4%; IQR, 92–97%), equal to the average ratio across national FBDG (Fig. 3a). The nutrients least likely to be adequate in least-cost diets meeting the HDB were calcium, vitamin B_12_, riboflavin, vitamin C (nutrient adequacy ratios, NAR = 77%, 82%, 85% and 86%). These are also the most limiting micronutrients in the national FBDG and EAT-Lancet (Fig. 3b). The HDB and national FBDG generally provide balanced macronutrient intakes, with protein at the low end and carbohydrate at the high end of acceptable macronutrient distribution ranges (AMDR) (Fig. 3d–f).

The HDB adheres well to World Health Organization (WHO) healthy diet recommendations related to prevention of noncommunicable diseases^28,29^. On average, the HDB met 8.7 (s.d., 1.0; IQR, 8–9) of 10 WHO recommendations, slightly higher than the average across national FBDG of 8.4 (s.d., 1.3; IQR, 8–9), and EAT-Lancet of 7.9 (s.d., 0.9; IQR, 7–9). The HDB diets consistently met recommendations on free sugar (100% of least-cost baskets met the recommendation), legumes, nuts and seeds (100%), processed meat (100%), red meat (99%) and salt (97%). The recommendations least likely to be met were on fibre (86%), total fat (84%), saturated fat (84%), fruits and vegetables (84%) and inclusion of whole grains (40%) (Fig. 3c). The volume of fruits and vegetables fell below the WHO recommendation of 400 g in cases where high-calorie items were cheapest, including avocados, bananas, immature coconut or dried fruits. The performance of the HDB and other FBDG in other diet quality scoring systems is shown in Supplementary Fig. 1.

**Fig. 4:**
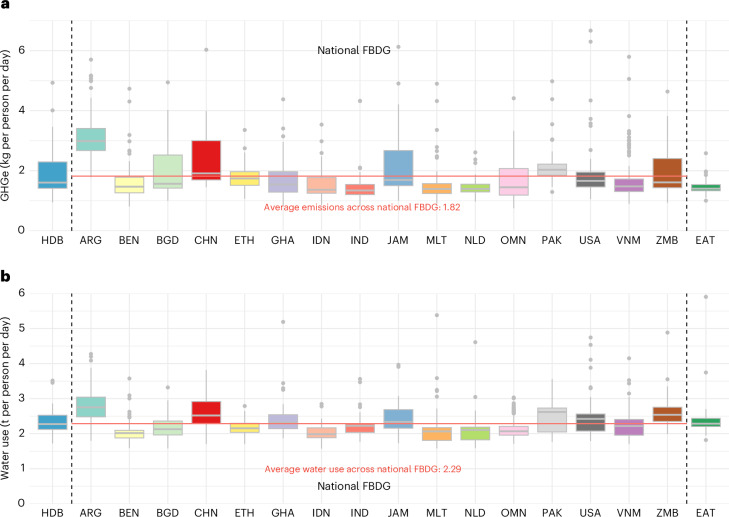
GHGe and water use of least-cost diets. **a**, GHGe (CO_2_ equivalent emissions in kg per person per day). The red solid line indicates the average value among all observations across national FBDG of 1.82 (median, 1.61), close to the HDB of 1.85 (median, 1.61) and slightly higher than the EAT-Lancet reference diet of 1.45 (median, 1.40). **b**, Water use in metric tons per person per day. The average of 2.29 (median, 2.21) across national FBDG shown in the red line is similar to 2.30 for HDB (median, 2.27) and 2.34 for EAT (median, 2.29). Box plots in both panels represent the IQR, with the horizontal line inside each box indicating the median. Whiskers extend to the smallest and largest observations within 1.5 times the IQR. Data points beyond this range are considered outliers and plotted individually.

Regarding environmental impact, the average greenhouse gas emissions (GHGe) of least-cost diets meeting the HDB were 1.85 kg per person per day (s.d., 0.61; IQR, 1.42–2.30), equivalent to the average across FBDG of 1.82 kg per person per day (s.d., 0.69; IQR, 1.36–2.05) and slightly higher than EAT-Lancet at 1.45 kg per person per day (s.d., 0.19; IQR, 1.34–1.54) (Fig. 4a). In comparison, the daily GHGe of current consumption globally is approximately 3.23 kg per person per day^30^. Average water use was 2.30 metric tons (t) per person per day (s.d., 0.29; IQR, 2.12–2.53), similar to EAT-Lancet at 2.34 t per person per day (s.d., 0.35; IQR, 2.20–2.44) and the average across FBDG of 2.29 t per person per day (s.d., 0.40; IQR, 2.01–2.50) (Fig. 4b).

## Discussion

This analysis shows that the HDB, and the national FBDG it is based on, encapsulate the elements of a healthy diet articulated in a joint statement by FAO and WHO^28^.

As a cost standard, the HDB results in a cost similar to other national FBDG and, importantly, does not overestimate cost, which would have led to an overestimate of the prevalence of unaffordability. The cost, and corresponding number of people unable to afford a healthy diet, would be slightly higher but in a similar range if individual national FBDG were used as the cost standard rather than the HDB. This is corroborated in ongoing work within countries to track the CoHD^31–35^. The HDB has been used to find subnational CoHD in countries that do not currently have national FBDG^33,36^.

The HDB represents well a variety of FBDG around the world, maintaining familiar food groupings in average amounts across countries. While FBDG differ in specifics and presentation, when quantified by daily food group recommendations they present a consistent picture of a healthy diet pattern, reflected in the HDB. Because it simply requires six food groups, the HDB allows for variation in food item preferences (that is, rice, maize, cassava or pasta can satisfy the starchy staple food requirement; no specific food items are specified). It requires animal-source foods but does not specify which, meaning that it allows for vegetarian diets and diets that restrict consumption of specific types of animal food based on religion (for example, beef or pork), and it does not require dairy, which is a common food in some cultures but is expensive and poorly digested in others. By including only the food groups common across countries, and taking the average amounts recommended across disparate countries and situations, idiosyncratic higher-cost requirements of specific countries’ FBDG (introduced by technical or political processes) lose their influence and the signal emerges through the noise. Rather than seeking to provide a new set of guidelines like the EAT-Lancet Commission, the HDB mirrors and amplifies what is already said by many. The HDB is politically attuned, reflecting UN member states’ own policy documents that define a healthy diet in context and the diligent work they encapsulate. Built from national FBDG, the HDB reveals an implicit consensus across countries on what a healthy diet is.

A key finding is that least-cost diets following the HDB have similar environmental impacts as the EAT-Lancet reference diet, a diet pattern widely recognized as sustainable. The HDB has a larger range of GHGe, owing to the substitutability of items with different GHGe within food groups. When applied in costing, the EAT-Lancet diet has more disaggregated food groups and therefore less substitutability. Although renowned for promoting a low-meat diet, EAT-Lancet has 13 food groups^37^—which, in costing exercises, paradoxically implies a daily minimum cost of red meat, fish, poultry and other specific food groups that are not required in FBDG nor in the HDB. The amount of red meat in the HDB could exceed the amount in EAT-Lancet, but in practice, red meat is rarely a least-cost item. Previous studies have looked at the environmental impact of FBDG given current consumption patterns, not least-cost selections^38,39^. Given that the HDB and EAT-Lancet have an almost identical water footprint, and the carbon footprint of both is about half that of current consumption patterns^30,39,40^, the HDB applied in CoAHD can be considered a healthy and sustainable diet.

The HDB also broadly meets micronutrient and macronutrient needs. A limitation of the HDB, like FBDG, is that each selected basket of items satisfying food group requirements does not necessarily meet 100% of micronutrient requirements. This is expected. FBDG are developed to meet nutrient needs on average, allowing day-to-day variation in food item selection and concomitant variation in nutrient intakes. Linear programming has been used for over two centuries to identify optimized least-cost diets that meet nutrient needs strictly within a 24 h period^41–44^; which is computationally practical but is not necessarily biological or social reality, producing results that often differ markedly from normal palatable diets or food group proportions of FBDG. Adding linear programming for food selection could solve for baskets of food items that precisely meet all nutrient requirements at 100%, but that type of analysis would deviate from tangible food group amounts that can be followed by regular people selecting foods in the market to meet recommendations for healthy diets. Episodic consumption of higher-cost foods rich in the limiting nutrients (calcium, vitamin B_12_, riboflavin, vitamin C) may require periodic additional expenditures beyond the daily CoHD to achieve nutrient adequacy over a time period longer than 24 h.

In some settings, wild foods such as dark green leafy vegetables contribute meaningfully to food access and nutrient adequacy^45,46^. A limitation of the CoAHD method is that it accounts for access based only on market prices. The food environment is not limited to formal markets where prices are routinely collected; it also includes informal markets, cultivated food, wild food, and gifts and transfers^47–49^. Retail market prices provide a good indicator of economic access, but solutions to unaffordability may go beyond markets and leverage own production or food transfers for increased access to healthy diets.

Another limitation is that the current CoAHD method does not explicitly account for food preferences—understood as the cultural preferences for food items that are necessary for a dignified diet (for example, in much of Asia, the staple of a meal is rice; a hypothetical least-cost diet including only sorghum and millet as starchy staples would not be acceptable). A food preferences variant of the CoHD uses all items for which prices are collected in proportion to their consumption shares, which overcomes this limitation but costs more^3^. It has been proposed by Mahrt et al. for poverty measurement that accounts for the CoHD as a basic need, in keeping with the principle of social inclusion^50^. In the future, countries could calculate CoHD restricting the list of items to only those with the highest consumption or expenditure shares as a proxy for cultural food preferences; they could also track the change in CoHD using consumer price indexes of each HDB food group, which would include all items weighted by expenditure shares. Expenditure shares are not available in the ICP price dataset, so they could not be used in global monitoring to date.

The approach of defining ‘sufficient food’ as a least-cost diet that meets FBDG is novel in food security measurement. ‘Food security’ often has the connotation of having enough food to meet energy needs. Indeed, this was how it was measured by FAO for 40 years, with prevalence of undernourishment (hunger) as the sole global food security indicator from 1974 until experience-based indicators were first measured in 2014. Analogously, the traditional standard for poverty measurement has been to find the cost of meeting energy needs, following the typical consumption patterns of the poor. Baskets resulting from an energy-only standard have been shown to be severely nutrient deficient, making it hard to argue that they are an acceptable standard of sufficient food^50^. The CoHD is consistently above $3 in PPP terms per person per day, whereas the extreme poverty line is $2.15, of which $1.35 can credibly be reserved for food after non-food basic needs are met—less than half of the CoHD^8^. This observation highlights that the current methodology for setting the poverty lines does not account for the CoHD. It should. The difference in cost between meeting energy needs, and meeting dietary needs, reveal that a significant portion of people identified as non-poor still cannot afford dietary needs for an active and healthy life.

Overall, 2.8 billion people cannot afford a healthy diet^7^. The majority who cannot afford the diet are in sub-Saharan Africa and South Asia. Even more are vulnerable to losing access to healthy diets due to shocks^51^, and during the global pandemic those who already could not afford a healthy diet became even less able to afford one^52^. Previous work defining the affordability of healthy versus unhealthy diets often examined the cost of actual consumption patterns, finding that people who consumed healthier diets tended to pay more for them than people who consumed unhealthy diets^53^. We also find this to be true worldwide, but based on prices, not preferences: the majority of people in low-income and lower-middle-income countries spend less on food than the least-cost healthy diet because they do not have sufficient income to afford a healthy diet.

The use of CoAHD represents a paradigm shift in the way ‘sufficient food’ is understood, operationalized and measured: from access to sufficient dietary energy, to access to healthy diets. This indicator, and a transparent methodology that reflects commonalities across countries’ own definitions of a healthy diet, have revealed a powerful view into the everyday lives of low-income people around the world, noticed acutely by mothers: that healthy diets are often unattainable. This is a problem that cannot be solved with nutrition education. It is a call to action to close the gap between economic investments in agriculture and the types of food most often recommended but most often missing in diets. It is a shift from the paradigm of sufficient food as calories to sufficient food as healthy diets^54^.

The HDB is a global standard consistent with all aspects of a healthy diet^28^ and the pillars of sustainability: the economic cost of a diet that meets needs for human health through dignified diets, while producing relatively low environmental impact. It is appropriate to use for comparing the cost and affordability of healthy and sustainable diets across countries. Even least-cost diets meeting this standard are unaffordable for many people in the world, pointing to the need for accelerated action to make healthy diets accessible for all.

## Methods

In this study, we calculated the cost and nutritional and environmental outcomes of a healthy diet by following a structured methodology using multiple dietary guidelines and national food price data. We began by identifying the specific requirements of various FBDG included in the analysis, including the HDB, national FBDG and the EAT-Lancet reference diet. Food price data were sourced from the ICP, a unique dataset that provides nationally representative average prices for a comprehensive range of food items across 173 countries in the most recent 2021 cycle. Each food item was matched with food composition tables to obtain calorie content and edible portion information, enabling conversion from price per kilogram to price per calorie. We used rank-order optimization to identify the lowest-cost items that meet the requirements of FBDG and calculated total diet costs in local currency, subsequently converting them to 2021 PPP dollars. Finally, we calculated both the nutritional and environmental outcomes for these least-cost healthy diets based on the quantities and prices of the selected foods. Statistical analyses were conducted using Stata MP18 and RStudio 2023.12.0 to assess variations in diet costs and affordability, nutritional indicators and environmental impacts.

### The HDB standard

The HDB comprises average recommended amounts of the most commonly recommended food groups in national FBDG: starchy staples, vegetables, fruits, protein-rich foods (both animal source and plant source) and oils/fats. The HDB was developed in 2022, from 10 quantified FBDG (the most recent within each major region) and 30 semi-quantified food guides from all regions of the world^9^. For the quantified FBDG, the recommended amounts of each food group were identified and converted from grams or heterogeneous servings into energy equivalents^55^. The total dietary energy was standardized to 2,330 kcal for comparability, and each food group recommendation was adjusted proportionally (Supplementary Table 1). Beyond countries where fully quantified guidelines were examined, many other countries have semi-quantified guidance that is shown pictorially in food guides (for example, plates, pyramids). Thirty countries were identified with semi-quantifiable food guides, which show food group proportionality approximately by volume, similar to the way food appears on a plate. The average recommended amounts in quantified guidelines were compared to the proportions shown in semi-quantitative food guides (Supplementary Table 2).

The dietary energy needs of an active 30-year-old woman, 2,330 kcal, were used as a standard energy requirement. This level of dietary energy is close to the median (2,328 kcal) and weighted mean (2,322 kcal) energy requirement of each sex, activity level and pregnancy and lactation status at each year of age (over 2 years)^10^. The dietary energy needs of a 30-year-old woman are therefore a reasonable representation of a generic total population. Previous research has demonstrated that least-cost diets to meet energy and nutrient requirements for people in this reference group are approximately the median level of costs for all sex–age groups over the entire life cycle^44^.

The average recommended proportions for each food group, standardized into caloric equivalents, form the HDB (Table 1). In terms of dietary energy, these are approximately one-half from starchy staples, one-quarter from protein-rich foods, one-eighth from vegetables and fruits, and one-eighth from oils and fats. By volume (appearance on a plate), these proportions correspond to approximately one-quarter starchy staples, one-quarter protein-rich foods, one-half fruits and vegetables, and a small proportion of added oils and fats (Extended Data Fig. 1 and Supplementary Table 3). These proportions reflect the modal volumetric proportions across all the plate-shaped food guides of countries around the world (Supplementary Table 2).

FBDG universally emphasize the need for variety within and between food groups^56^. For its function as a cost standard, the HDB operationalizes the construct of variety by specifying the number of least-cost items in each food group as two fruits; three vegetables; two starchy staples; two animal-source foods; one legume, nut or seed; and one oil or fat: 11 items in total (Table 1). The total number aligns FBDG that include a recommendation on number of foods to consume in 1 day; for example, China’s FBDG aim for 12 different individual foods or more in a day.

### FBDG quantification

In this study, we use FBDG from Argentina, Bangladesh, Benin, China, Ethiopia, Ghana, Indonesia, India, Jamaica, Malta, Netherlands, Oman, Pakistan, the United States, Viet Nam and Zambia^12–27^. They were accessed primarily through the FAO FBDG repository, and all materials were available in English, Spanish and/or French^57^. As a reference for a diet pattern with low environmental impact, the EAT-Lancet reference diet was also quantified for comparison with the HDB, using the mean reference amounts of each food group^11^. The detailed quantification of each FBDG is publicly available^55^.

### Diet cost analysis

The CoHD is computed by classifying food items into specified food groups, calculating the cost per day of each retail item in quantities required to meet energy targets and using rank-order optimization to select lowest-cost items in each food group. The cost per day of a retail food item, *i*, is based on its price per kilocalorie, *p*_*i*_, multiplied by the quantity, *q*_*i*_, required to meet the relevant energy target within a food group. Food group costs are then calculated by summing the cost per day of lowest-cost items selected into each food group, as follows, using the HDB food groups and energy targets:

1 $$\begin{array}{l}{\rm{Cost}}_{\rm{StarchyStaples}}=\min\left\{\mathop{\sum}\limits_{i=1}^{2}{p}_{i}{q}_{i}\right\},\,{\rm{where}}\,{\rm{each}}\,{q}_{i}\\\qquad\qquad\qquad\quad=580\,{\rm{kcal}}\,{\left(=\frac{1,\!160}{2}\right)}\;{\rm{and}}\,{\rm{item}}\,i\,{\rm{is}}\,{\rm{a}}\,{\rm{starchy}}\,{\rm{staple}}\,\end{array}$$

2 $$\begin{array}{l}{\rm{Cost}}_{\rm{AnimalFoods}}=\min\left\{\mathop{\sum}\limits_{i=1}^{2}{p}_{i}{q}_{i}\right\},{\rm{where}}\,{\rm{each}}\,{q}_{i}=150\,{\rm{kcal}}\,\left(=\frac{300}{2}\right)\\\quad\;{\rm{and}}\,{\rm{item}}\,i\,{\rm{is}}\,{\rm{an}}\,{\rm{animal}}\,{\rm{source}}\,{\rm{food}}\,\end{array}$$

3 $$\begin{array}{l}{\rm{Cost}}_{\rm{LegsNutsSeeds}}=\left\{\,{p}_{i}{q}_{i}\right\}\,,{\rm{where}}\,{\rm{each}}\,{q}_{i}\\\qquad\qquad\qquad\quad=300\,{\rm{kcal}}\,{\rm{and}}\,{\rm{item}}\,i\,{\rm{is}}\,{\rm{a}}\,{\rm{legume}},\,{\rm{nut}}\,{\rm{or}}\,{\rm{seed}}\,\end{array}$$

4 $$\begin{array}{l}{\rm{Cost}}_{\rm{Vegetables}}=\min\left\{\mathop{\sum}\limits_{i=1}^{3}{p}_{i}{q}_{i}\right\},{\rm{where}}\,{\rm{each}}\,{q}_{i}\\\qquad\qquad\quad\;\;\,=66.7\,{\rm{kcal}}\,\left(=\frac{110}{3}\right)\;{\rm{and}}\,{\rm{item}}\,i\,{\rm{is}}\,{\rm{a}}\,{\rm{vegetable}}\,\end{array}$$

5 $$\begin{array}{l}{\rm{Cost}}_{\rm{Fruits}}=\min\left\{\mathop{\sum}\limits_{i=1}^{2}{p}_{i}{q}_{i}\right\},{\rm{where}}\,{\rm{each}}\,{q}_{i}\\\qquad\qquad\;=80\,{\rm{kcal}}\,\left(=\frac{160}{2}\right)\;{\rm{and}}\,{\rm{item}}\,i\,{\rm{is}}\,{\rm{a}}\,{\rm{fruit}}\,\end{array}$$

6 $${\rm{Cost}}_{\rm{OilsFats}}=\{\,{p}_{i}{q}_{i}\}\,,\,{\rm{where}}\,{\rm{each}}\,{q}_{i}=300\,{\rm{kcal}}\,{\rm{and}}\,{\rm{item}}\,i\,{\rm{is}}\,{\rm{a}}\,{\rm{fat}}\,$$

The total CoHD is calculated by summing costs across food groups, as follows:

7 $${\rm{Cost}}_{\rm{HealthyDiet}}=\mathop{\sum}\limits_{j=1}^{6}{\rm{Cost}}_{j},\,{\rm{for}}\,{\rm{all}}\,j=\{1,\,\ldots ,\,6\}\,{\rm{food}}\,{\rm{groups}}$$

The food groups and energy targets in the above set of equations are adjusted for each national FBDG when computing the lowest cost of meeting each FBDG, and the EAT-Lancet reference diet^55^.

To compare diet costs across countries, we used retail price data reported by national statistical agencies through the ICP, managed by the World Bank as part of a global statistical collaboration under the United Nations Statistical Commission^58^. The ICP collects prices for standardized items worldwide to compute PPP. For this study, we used 2021 ICP prices, the latest available round. These data provide annual average, seasonally adjusted and nationally representative prices in local currency units for 735 foods and non-alcoholic beverages across 173 countries.

For the diet cost analysis following the HDB, 562 items were used. Excluded items included those that were non-caloric, ingredients, condiments, baby food, items with unclear composition and foods not recommended as part of a healthy diet, such as trans fats, sugar-sweetened beverages and processed meats. The dataset provides extensive food item coverage across countries, with an average of 105 priced food items per country. Detailed information on item numbers used for the calculation of CoHD in each HDB food group can be found in Supplementary Table 4.

The variation in the number of countries included in the analysis for each dietary guideline is due to the availability of price data. FBDG may require specific food groups, and in some countries, price data for items within those groups may not be available. This results in slight differences in the number of countries analysed across the various diets. Data were then available from 172 countries to calculate the cost of meeting the FBDG of Argentina, Benin, Malta, Pakistan, the United States and Viet Nam; 171 countries for the HDB, FBDG of Bangladesh, China, Ghana, India, Indonesia, Jamaica, the Netherlands and Zambia; 169 countries for FBDG of Ethiopia and Oman; and 162 countries for EAT-Lancet as it requires the most specific food groups.

The diet cost estimation in local currency units were then converted to 2021 PPP dollars using the latest PPP conversion factors for households and non-profit institutions serving households final consumption expenditure (previously termed private consumption). This approach differs slightly from the State of Food Security and Nutrition in the World 2024 report, which estimates the CoHD based on 2017 PPP prices^7^.

We then compared the CoHD following the HDB with GNI per capita per day and food expenditures per capita per day, by country, sourced from national accounts data on household food expenditures assembled by the World Bank^59^.

### Analysis of nutrient content and other nutritional characteristics

The macro- and micronutrient content of the least-cost diets meeting criteria for the HDB, 16 national FBDG and the EAT-Lancet reference diet were analysed for up to 172 countries. For macronutrients, we examined whether the proportions of carbohydrate, fat and protein fall within the AMDR^60^. For 15 micronutrients and protein, we apply global harmonized average requirements, which are the levels of nutrients that meet the needs of 50% of the healthy population of each age and sex^61^. Beyond nutrients, we assessed the extent to which the diets met global healthy diet recommendations related to protection of health against noncommunicable diseases. These include recommendations on dietary components to include in healthy diets (at least 400 g fruits and vegetables and at least 25 g fibre daily, and consumption of whole grains, legumes, nuts and seeds) and to limit in healthy diets (excessive sugar (<10% dietary energy), salt (<5 g d^−1^), total fat (≤30% dietary energy), saturated fat (<10% dietary energy), little if any processed meat and red meat (no more than 350–500 g week^−1^, or 71 g d^−1^))^28,29^. They are summarized as a simple count of the number of WHO dietary recommendations met. The performance of the HDB and other FBDG in other diet quality scoring systems, the Alternative Healthy Eating Index and Diet Quality Index-International was also assessed in auxiliary analyses^62,63^.

### Analysis of environmental impact

To examine environmental impact, we calculated the GHGe (in kg CO_2_eq per person per day) and water use (litres per person per day) of the least-cost diets that met the HDB, FBDG and EAT-Lancet criteria. Food items in the ICP food price list were matched to food items in a database of carbon and water footprints^64^. Items without an exact match were matched to the closest item within the same food group, in terms of biological similarity (for example, same species, genus or family) and edible portion. Then the GHGe and water use of each least-cost diet were calculated for up to 172 countries.

### Statistics and reproducibility

This study uses nationally representative food price datasets across 173 countries. No statistical method was used to predetermine sample size. Countries were omitted only when necessary due to missing food price or other required data. As this study is based on secondary data analysis of national datasets, randomization and blinding were not applicable. Investigators were not blinded to data allocation during analysis and outcome assessment. The methodology follows standardized approaches for diet cost estimation and nutritional and environmental assessment, ensuring consistency and reproducibility.

### Reporting summary

Further information on research design is available in the Nature Portfolio Reporting Summary linked to this article.

## Supplementary information

Supplementary Information Supplementary Fig. 1 and Tables 1–4.

Reporting Summary

## Data Availability

Food item descriptions and prices for this study were used under a confidentiality agreement with the ICP^58^ and are not publicly available. Researchers seeking access to these data should refer to the ICP data access policy at https://www.worldbank.org/en/programs/icp/data. The dataset on carbon and water footprints of food commodities is publicly available and can be accessed from Petersson et al.^64^. The food composition data are publicly available via the USDA Food Data Central at https://fdc.nal.usda.gov.

## Code availability

Replication code for producing the results is available at 10.17605/OSF.IO/47D3M. Additional scripts supporting the findings of this study can be provided upon reasonable request.
