# Supplementary material for: The Healthy Diet Basket is a valid global standard that highlights lack of access to healthy and sustainable diets
Source: Nat Food. 2025 May 27;6(6):622–31. doi: 10.1038/s43016-025-01177-0 (PMC12185308; doi:10.1038/s43016-025-01177-0)
Supplement: Supplementary file 1 — Supplementary Fig. 1 and Tables 1–4. [file 43016_2025_1177_MOESM1_ESM.pdf]

nature food

Supplementary information https://doi.org/10.1038/s43016-025-01177-0

The Healthy Diet Basket is a valid global standard that highlights lack of access to healthy and sustainable diets

In the format provided by the authors and unedited

The Healthy Diet Basket is a valid global standard that highlights lack of access to healthy and sustainable diets

Anna W. Herforth[1*], Yan Bai[2,3*], Aishwarya Venkat[1], William A. Masters[1]

1 Food Prices for Nutrition Project, Friedman School of Nutrition Science and Policy, Tufts University, Boston, MA

2 Development Data Group, the World Bank, Washington, DC

3 School of Public Affairs, Zhejiang University, Hangzhou, China

Contact: anna@annaherforth.net and ybai@worldbank.org

Supplementary Information

Section I. Supplementary Figures

Figure S1. Assessment of diet quality scores of least-cost diets by food-based dietary guideline (FBDG), across 162-172 countries

Section II. Supplementary Tables

Table S1. Average food group amounts recommended across FBDG scaled to meet a consistent dietary energy intake target (2 330 kcal)

Table S2. Food group proportions (by volume) depicted in plate-shaped Food Guides from all countries where they were available

Table S3. Healthy Diet Basket content by food group: Average food group amounts recommended across FBDG scaled to meet a consistent dietary energy intake target (2 330 kcal), translated into gram amounts by reference food items

Table S4. Average item number of food items qualified for CoHD calculation across HDB food groups by World Bank region and income classification, 2021

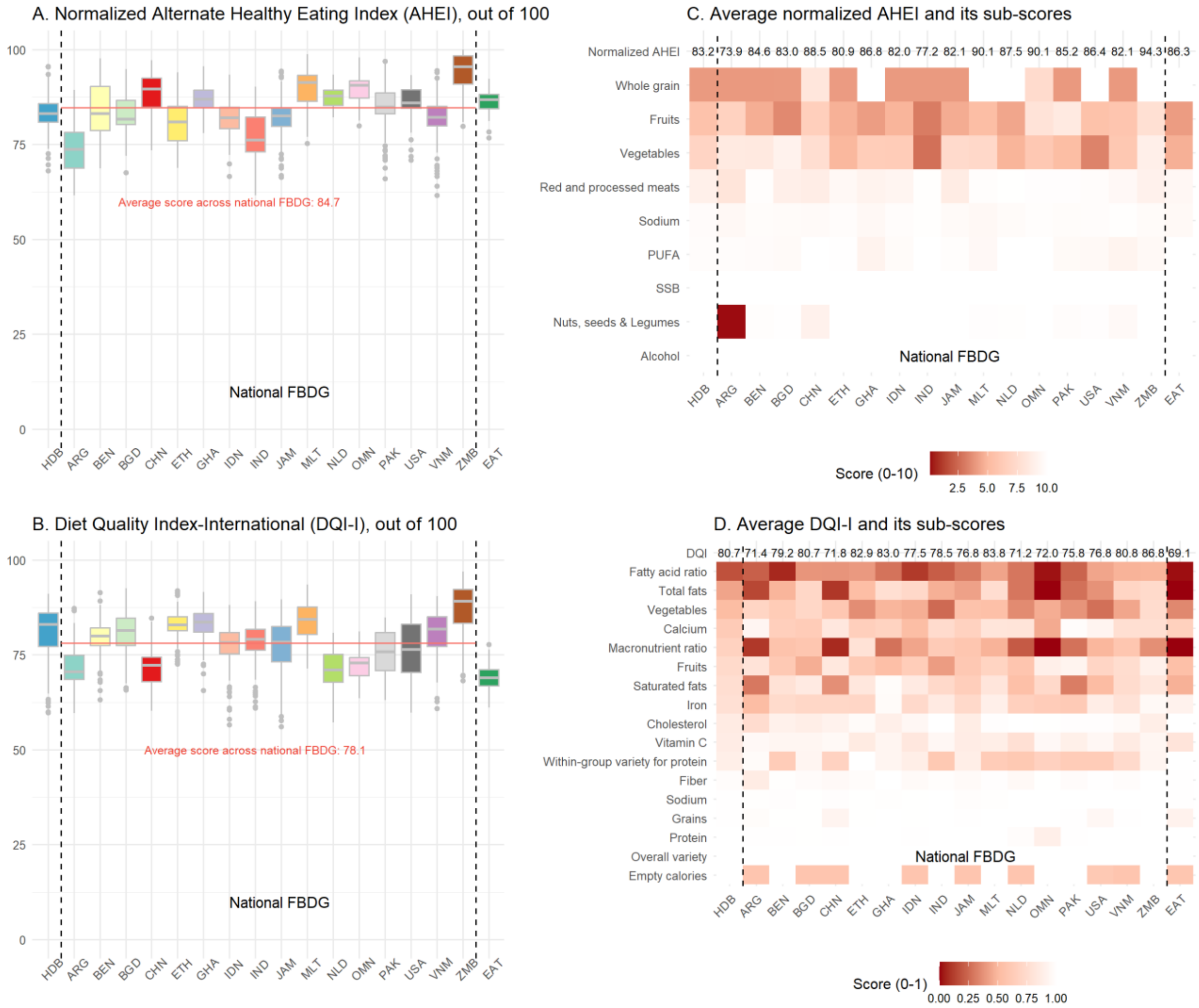

Figure S1. Assessment of diet quality scores of least-cost diets by food-based dietary guideline (FBDG) across 162-172 countries. (A) illustrates the normalized Alternate Healthy Eating Index (AHEI) scores distribution across various FBDG, represented through box plots. The AHEI is calculated based on 9 dietary components, including fruits, vegetables, nuts & seeds & legumes, whole grains, sugar-sweetened beverages (SSBs), polyunsaturated fatty acids (PUFAs), red and processed meat, sodium, and alcohol. Each component's score ranges from 0 to 10, with higher scores indicating better adherence to healthy eating guidelines. Scores of all components are summed, and the total score is normalized to a percentage point out of 100. (B) depicts the Diet Quality Index-International (DQI-I) scores across different FBDG. The DQI-I comprises four categories, encapsulating a total of 17 components. These categories are variety, adequacy, moderation, and overall balance, evaluating the diet's diversity, nutrient sufficiency, moderation in consumption of certain nutrients and foods, and balanced nutrient ratios. (C) presents a heatmap for assessing the scores of the AHEI's 9 dietary components across the various FBDG. (D) features a heatmap for the DQI-I's 17 components, normalized between 0 and 1 for comparative purposes. The AHEI contained criteria that could not be analyzed from the level of specificity available in the food price data, including omega-3 fatty acids and trans-fat. Box plots in (A) and (B) represent the interquartile range (IQR), with the horizontal line inside each box indicating the median. Whiskers extend to the smallest and largest observations within 1.5 times the IQR. Data points beyond this range are considered outliers and plotted individually.

Section II. Supplementary Tables

Table S1. Average food group amounts recommended across FBDG scaled to meet a consistent dietary energy intake target (2,330 kcal)[1]

	**Starchy staples**		**Vegetables**		**Fruit**		**Protein-rich foods[2]**		**Oils**	
**FBDG**	kcal	grams	kcal	grams	kcal	grams	kcal	grams	kcal	grams
**Argentina**	1 000	278	145	482	228	362	638	446	320	36
**Benin**	1 216	338	133	443	167	266	462	323	352	40
**China**	1 238	344	117	391	169	269	568	398	237	27
**India**	1 009	280	123	409	110	175	809	566	279	32
**Jamaica**	1 162	323	117	389	130	206	630	441	291	33
**Malta**	1 343	373	112	375	148	234	572	400	155	18
**Netherlands**	1 205	335	74	247	124	197	577	404	349	39
**Oman**	942	262	101	337	370	587	335	234	583	66
**United States of America**	1 038	288	89	297	187	297	753	527	263	30
**Viet Nam**	1 165	324	92	307	166	263	640	447	267	30
**Mean**	1 132	314	110	368	180	286	598	422	310	35
**Median**	1 164	323	114	382	167	264	604	419	285	32
**HDB**	1 160	322	110	367	160	254	600	420	300	34

Notes:

1. Data shown are authors' calculations from national government documents. Quantified recommendations from these ten national FBDG represent a range of dietary recommendations articulated by countries in each region. An updated set of links to country websites is available from the FAO at https://www.fao.org/nutrition/education/food-dietary-guidelines. Grams are based on kcal equivalent of all foods in the group to reference food items: egg for protein-rich foods and dry rice for starchy staples. Calories per gram of fruits and vegetables, respectively, are based on the average across all

non-duplicate fruits (0.63 kcal/g) and vegetables (0.3 kcal/g) in the 2017 ICP dataset. HDB = Healthy Diet Basket. This table originally appeared in Herforth et al., 2022.

2. Protein-rich foods here combine dairy with other protein-rich foods, including meat, fish, egg, legumes, and/or nuts and seeds. The median amount for dairy across the 10 FBDG was 230 kcal, a food group present in all the selected FBDG except Jamaica's. In some countries, protein-rich food group has sub-categories: Argentina: (a) meat, fish, egg; (b) dairy; Benin: (a) meat, fish, egg, legumes, nuts and seeds; (b) dairy; China: (a) meat, fish, egg; (b) dairy; (c) soy, nuts, seeds; India: (a) meat, fish, egg, legumes; (b) nuts and seeds; (c) dairy; Jamaica: (a) foods from animals including dairy; (b) legumes and nuts; Malta: (a) meat, fish, eggs, legumes, nuts and seeds; (b) dairy; Netherlands: (a) meat, fish, egg, legumes; (b) nuts and seeds; (c) dairy; Oman: (a) meat, fish, egg, nuts and seeds; (b) legumes; (c) dairy; United States of America: (a) meat, fish, egg, legumes, nuts and seeds; (b) dairy; Viet Nam: (a) meat, fish, egg, legumes; (b) dairy.

Table S2. Food group proportions (by volume) depicted in plate-shaped Food Guides from all countries where they were available[1]

Country/year	Starchy staples	Vegetables	Fruits	Vegetables and fruits	Protein-rich foods[2]	Fats and oils	Total
Antigua and Barbuda (2013)	39	14	14	28	28	5	100
Argentina (2015)	25			50	20	5	100
Australia (2013)	30	28	16	44	26	0	100
Canada (2019)	25			50	25	0	100
Chile (2013)	15	30	20	50	30	5	100
Colombia (2015)	30			27	38	5	100
Domenica (2007)	28	25	17	42	25	5	100
Ecuador (2018)	25			50	20	5	100
Germany (2017)	30	25	17	42	25	3	100
Grenada (2006)	47	10	10	20	27	6	100
Guyana (2018)	33	15	15	30	30	7	100
India (2018)	27			50	20	3	100
Italy (2018)	20	27	27	54	20	6	100
Jamaica (2015)	30	24	25	49	18	3	100
Latvia (2008)	25			50	25	0	100
Malawi (unofficial)	33	23	22	45	17	5	100
Malta (2015)	25	27	20	47	25	3	100
Mexico (2015)	33	16	16	32	35	0	100
Oman (2009)	34	15	15	30	36	0	100
Pakistan (2018)	33	19	15	34	27	6	100
Panama (2013)	38	16	16	32	25	5	100
Peru (2019)	36	16	16	32	27	5	100
Poland (2020)	25			50	25	0	100
Portugal (2003)	27	24	20	44	26	3	100
Qatar (2015)	27	25	13	38	35	0	100
South Korea (2015)	30	24	16	40	30	0	100

Switzerland (2011)	40			40	20	0	100
United Kingdom of Great Britain and Northern Ireland (2016)	37			37	24	2	100
United States of America (2020)	25	30	20	50	25	0	100
Uruguay (2016)	28	30	20	50	16	6	100
Zambia (2021)	34	15	18	33	33	0	100
Mean	30.1	21.7	17.6	41.0	25.9	3.0	100
Median	**30.0**	24.0	16.5	**42.0**	**25.0**	**3.0**	100
Mode	**25.0**	30.0	20.0	**50.0**	**25.0**	**0.0**	100
HDB	**25.3%**	28.8%	19.9%	**48.8%**	**23.3%**	**2.7%**	100%

Notes:

1. Proportions (by volume) of major food groups displayed in plate-shaped dietary guides from 30+ countries. Data collected via visual inspection and scaled to 100%, accessed March 2022 at: https://www.fao.org/nutrition/education/food-based-dietary-guidelines. This table originally appeared in Herforth et al., 2022.
2. Protein-rich foods include dairy, other animal source foods, legumes, and sometimes nuts and seeds.

Table S3. Healthy Diet Basket content by food group: Average food group amounts recommended across FBDG scaled to meet a consistent dietary energy intake target (2 330 kcal), translated into gram amounts by reference food items

Food group	Total energy content (kcal)	Equivalent gram content, by reference food[1] (edible portion)
Starchy staples	1 160	322 g dry rice
Vegetables	110	270–400 g vegetables
Fruits	160	230–300 g fruits
Animal source foods	300	210 g egg
Legumes, nuts & seeds	300	85 g dry bean
Oils & fats	300	34 g oil

Note:

1. Different food items have different calories per gram. To equate calories and grams, the following reference food items were used: dry rice for starchy staples; egg for animal source foods; dry bean for legumes, nuts and seeds; and average kcal/g across all non-duplicate items in the 2017 ICP dataset for fresh fruits and vegetables.

Table S4. Average item number of food items qualified for CoHD calculation across HDB food groups by World Bank region and income classification, 2021

Name	Animal-source food	Fruits	Legumes, nuts and seeds	Fats and oils	Starchy Staples	Vegetables	Total count
**Income classification, World Bank, 2021**							
High income countries	34	8	6	6	24	11	89
Upper-middle countries	42	10	7	6	27	12	105
Lower-middle countries	53	11	8	8	31	13	125
Low income countries	52	10	7	8	29	13	119
**Region, World Bank**							
East Asia & Pacific	51	10	7	7	31	15	122
Europe & Central Asia	41	9	6	6	26	11	100
Latin America & Caribbean	28	8	5	6	21	10	77
Middle East & North Africa	49	16	11	7	35	15	133
North America	26	6	4	6	20	8	69
South Asia	42	11	9	7	36	14	118
Sub-Saharan Africa	51	10	7	8	28	12	115
**World**	**43**	**10**	**7**	**7**	**27**	**12**	**106**

Note:
This table shows the number of food items which are qualified for the Cost of a Healthy Diet (CoHD) calculation for 2021, categorized by six Healthy Diet Basket (HDB) food groups across World Bank income classifications and regional groupings. The global average ("World") reflects all 171 countries with available data. Anguilla, Bonaire, and Montserrat are excluded from income classification and regional results due to the absence of corresponding regional and income classification information in the World Bank datasets.
