# Supplementary material for: The Healthy Diet Basket is a valid global standard that highlights lack of access to healthy and sustainable diets
Source: Nat Food. 2025 May 27;6(6):622–31. doi: 10.1038/s43016-025-01177-0 (PMC12185308; doi:10.1038/s43016-025-01177-0)
Supplement: Supplementary file 2 — Reporting Summary [file 43016_2025_1177_MOESM2_ESM.pdf]

nature portfolio

Corresponding author(s): Anna W. Herforth and Yan Bai

Last updated by author(s): Mar 26, 2025

Reporting Summary

Nature Portfolio wishes to improve the reproducibility of the work that we publish. This form provides structure for consistency and transparency in reporting. For further information on Nature Portfolio policies, see our Editorial Policies and the Editorial Policy Checklist.

Statistics

For all statistical analyses, confirm that the following items are present in the figure legend, table legend, main text, or Methods section.

n/a	Confirmed	
☐	☒	The exact sample size (*n*) for each experimental group/condition, given as a discrete number and unit of measurement
☐	☒	A statement on whether measurements were taken from distinct samples or whether the same sample was measured repeatedly
☒	☐	The statistical test(s) used AND whether they are one- or two-sided<br>*Only common tests should be described solely by name; describe more complex techniques in the Methods section.*
☒	☐	A description of all covariates tested
☒	☐	A description of any assumptions or corrections, such as tests of normality and adjustment for multiple comparisons
☐	☒	A full description of the statistical parameters including central tendency (e.g. means) or other basic estimates (e.g. regression coefficient) AND variation (e.g. standard deviation) or associated estimates of uncertainty (e.g. confidence intervals)
☒	☐	For null hypothesis testing, the test statistic (e.g. *F*, *t*, *r*) with confidence intervals, effect sizes, degrees of freedom and *P* value noted<br>*Give P values as exact values whenever suitable.*
☒	☐	For Bayesian analysis, information on the choice of priors and Markov chain Monte Carlo settings
☒	☐	For hierarchical and complex designs, identification of the appropriate level for tests and full reporting of outcomes
☒	☐	Estimates of effect sizes (e.g. Cohen's *d*, Pearson's *r*), indicating how they were calculated

Our web collection on statistics for biologists contains articles on many of the points above.

Software and code

Policy information about availability of computer code

Data collection	Secondary data analysis with no software was used for data collection.
Data analysis	The data analysis is conduced using Stata MP18 and RStudio 2023.12.0 for data visualization.

For manuscripts utilizing custom algorithms or software that are central to the research but not yet described in published literature, software must be made available to editors and reviewers. We strongly encourage code deposition in a community repository (e.g. GitHub). See the Nature Portfolio guidelines for submitting code & software for further information.

Data

Policy information about availability of data

All manuscripts must include a data availability statement. This statement should provide the following information, where applicable:

- Accession codes, unique identifiers, or web links for publicly available datasets
- A description of any restrictions on data availability
- For clinical datasets or third party data, please ensure that the statement adheres to our policy

The underlying food price data used in this study are sourced from the International Comparison Program (ICP) and can be accessed according to the data access policy available at https://www.worldbank.org/en/programs/icp/data. The food composition data are publicly available from the USDA Food Data Central, supplemented with food composition tables from FAO and other countries. The food environmental data are from the study by Petersson et al. titled "A multilevel carbon and water footprint dataset of food commodities," published in Scientific Data (2021).

Research involving human participants, their data, or biological material

Policy information about studies with human participants or human data. See also policy information about sex, gender (identity/presentation), and sexual orientation and race, ethnicity and racism.

Reporting on sex and gender	NA
Reporting on race, ethnicity, or other socially relevant groupings	NA
Population characteristics	NA
Recruitment	NA
Ethics oversight	NA

Note that full information on the approval of the study protocol must also be provided in the manuscript.

Field-specific reporting

Please select the one below that is the best fit for your research. If you are not sure, read the appropriate sections before making your selection.

☐ Life sciences ☐ Behavioural & social sciences ☒ Ecological, evolutionary & environmental sciences

For a reference copy of the document with all sections, see nature.com/documents/nr-reporting-summary-flat.pdf

Ecological, evolutionary & environmental sciences study design

All studies must disclose on these points even when the disclosure is negative.

Study description	This study uses the Healthy Diet Basket (HDB) to compare the cost, nutritional adequacy, and environmental impact of healthy diets against 16 national dietary guidelines and the EAT-Lancet reference diet. Data from 162-172 countries in 2021 were analyzed to calculate the least-cost diets meeting these guidelines, providing insights into global diet affordability and sustainability.
Research sample	The research sample includes food price data from 162-172 countries collected in 2021. The study uses the least-cost diets meeting 16 national food-based dietary guidelines and the EAT-Lancet reference diet to analyze the cost, nutritional adequacy, and environmental impact.
Sampling strategy	The sampling strategy involved collecting nationally representative retail food price data from the International Comparison Program (ICP) for 2021. Data for 172 countries are available for our study.
Data collection	Food pirce data were collected from the International Comparison Program (ICP) for 2021. The ICP collects national annual average prices for a basket of goods and services to produce Purchasing Power Parities (PPPs). Additional data were sourced from the USDA Food Data Central, supplemented with food composition tables from FAO and other countries, and the multilevel carbon and water footprint dataset by Petersson et al. (2021).
Timing and spatial scale	The study uses data from the year 2021. The spatial scale of the data collection encompasses up to 172 economies globally.
Data exclusions	The study excluded non-caloric ingredients, condiments, baby food, and items not recommended as part of a healthy diet, such as trans fats and processed meats, from the dataset.
Reproducibility	All attempts to repeat results are successful.
Randomization	Randomization was not applicable in this study. The data collection involved obtaining nationally representative retail food price data from the International Comparison Program (ICP) and other publicly available sources, without the need for random assignment.
Blinding	Blinding was not applicable in this study. The research involved the analysis of publicly available data on food prices, composition, and environmental impact, which did not require blinding of participants or researchers.

Did the study involve field work? ☐ Yes ☒ No

Reporting for specific materials, systems and methods

We require information from authors about some types of materials, experimental systems and methods used in many studies. Here, indicate whether each material, system or method listed is relevant to your study. If you are not sure if a list item applies to your research, read the appropriate section before selecting a response.

Materials & experimental systems

n/a	Involved in the study
☒	☐ Antibodies
☒	☐ Eukaryotic cell lines
☒	☐ Palaeontology and archaeology
☒	☐ Animals and other organisms
☒	☐ Clinical data
☒	☐ Dual use research of concern
☒	☐ Plants

Methods

n/a	Involved in the study
☒	☐ ChIP-seq
☒	☐ Flow cytometry
☒	☐ MRI-based neuroimaging

Plants

Seed stocks	NA
Novel plant genotypes	NA
Authentication	NA
